# Synthesis and Comparative Electrochemical Evaluation of Polymer Cobalt Phthalocyanine and SWCNT-Modified Composite as High-Performance Anode Materials for Lithium-Ion Batteries

**DOI:** 10.3390/polym18161936

**Published:** 2026-08-07

**Authors:** Keshavananda Prabhu Channabasavana Hundi Puttaningaiah, Ashwini Chikkabasur Kumbara, Jaehyun Hur

**Affiliations:** 1 School of Chemical, Biological, and Battery Engineering, Gachon University, Seongnam-si 13120, Gyeonggi-do, Republic of Korea; 2 Department of Chemistry, Kishkinda University, Mount View Campus, Ballari 583120, Karnataka, India

**Keywords:** polymer cobalt phthalocyanine, SWCNT, lithium-ion batteries, anode materials, electrochemical performance, energy storage, composite materials, charge transfer, cycling stability

## Abstract

The development of high-performance anode materials remains a key challenge for advancing lithium-ion battery (LIB) technology. In this work, an oxy-bridged polymer cobalt phthalocyanine (Poly-CoPc) and its single-walled carbon nanotube-modified composite (Poly-CoPc/SWCNT) were successfully synthesized and systematically investigated as potential anode materials. The structural, chemical, and morphological properties of the materials were thoroughly characterized using Fourier-transform infrared spectroscopy (FT-IR), Raman spectroscopy, X-ray diffraction (XRD), Brunauer–Emmett–Teller (BET) surface area analysis, scanning electron microscopy (SEM), energy-dispersive X-ray spectroscopy (SEM-EDX), and transmission electron microscopy (TEM) techniques. Morphological studies demonstrated a uniform dispersion of Poly-CoPc over the SWCNT network, leading to an interconnected conductive structure with enhanced surface area. Electrochemical performance was evaluated and compared with pristine Poly-CoPc. Compared to pristine Poly-CoPc, the Poly-CoPc/SWCNT composite exhibited significantly improved electrochemical behavior, including higher specific capacity, enhanced rate performance, and superior cycling stability. The composite delivered a high initial discharge capacity of 2016 mA g^−1^ and maintained an excellent reversible capacity of 1047 mA g^−1^ after 100 cycles at 0.1 A g^−1^. Furthermore, it demonstrated outstanding rate capability, retaining a capacity of 855 mA g^−1^ at 0.5 A g^−1^. This enhanced performance is attributed to the synergistic effect between the redox-active Poly-CoPc and the highly conductive SWCNT network, which facilitates efficient electron transport, improves ion diffusion, and stabilizes the electrode structure during cycling. These results highlight that SWCNT-modified Poly-CoPc is a promising candidate for next-generation high-performance LIB anodes.

## 1. Introduction

The rapid expansion of portable electronics, electric vehicles, and large-scale energy storage systems has significantly increased the demand for high-performance lithium-ion batteries (LIBs) [1,2]. Although LIB technology has matured over the past few decades, the development of next-generation systems with higher energy density, faster charging capability, and longer cycle life remains a major challenge [3]. Currently, graphite is widely used as the commercial anode material due to its excellent stability and low working potential [4]. However, its relatively low theoretical capacity (~372 mAh g^−1^) and limited rate capability restrict further improvements in battery performance, particularly for high-power and fast-charging applications. These limitations have motivated extensive research into alternative anode materials with enhanced electrochemical properties. Various classes of materials, including transition metal oxides, sulfides, and alloy-based systems, have been investigated as potential anodes [5,6]. While many of these materials offer higher theoretical capacities, they often suffer from serious drawbacks such as large volume expansion during cycling, poor electrical conductivity, and rapid capacity fading. In this context, organic and metal–organic materials have emerged as promising candidates due to their structural diversity, lightweight nature, and tunable electrochemical properties [7,8,9,10,11]. Among them, metal phthalocyanines (MPcs) have attracted increasing attention because of their unique conjugated macrocyclic structure and redox-active metal centers [12,13,14].

Metal phthalocyanines exhibit several advantageous features, including high chemical and thermal stability, strong π–π conjugation, and the ability to undergo reversible redox reactions. Importantly, their electronic structure can be tailored by varying the central metal ion or by introducing functional substituents on the macrocycle, offering significant flexibility in designing materials with desired properties [15,16]. Recent studies have demonstrated the thermal robustness of MPc-based materials under high-temperature conditions, further supporting their suitability for electrochemical energy storage applications [17]. Similarly, other studies have reported that MPc-based materials can deliver moderate to high capacities in LIB systems, typically in the range of ~600–900 mAh g^−1^ depending on the metal center, morphology, and electrode architecture [18,19]. However, their practical application is still hindered by intrinsic limitations such as low electrical conductivity and dissolution or structural instability during repeated lithiation/delithiation cycles. Among various MPcs, cobalt phthalocyanine (CoPc) has gained particular interest due to the redox activity of the cobalt center and its relatively stable coordination environment. CoPc-based materials have been explored in a wide range of applications, including electrocatalysis (e.g., hydrogen/oxygen evolution and reduction and CO_2_ reduction reactions), sensors, supercapacitors, and energy storage devices [20,21,22]. In LIBs, CoPc can participate in reversible lithium (Li) storage through both ligand-centered and metal-centered redox processes. Nevertheless, pristine CoPc typically exhibits poor cycling stability and limited rate performance due to sluggish charge transfer kinetics and the aggregation of molecules, which reduce the number of accessible active sites.

To overcome these challenges, several strategies have been proposed, such as polymerization, nanostructuring, and hybridization with conductive carbon materials [23,24]. The polymerization of CoPc is particularly effective in enhancing structural stability by forming extended networks that suppress dissolution and improve mechanical integrity. However, even polymerized CoPc may still suffer from insufficient electrical conductivity, which limits its full electrochemical utilization. In this regard, the incorporation of highly conductive carbon nanomaterials, especially single-walled carbon nanotubes (SWCNTs), offers a promising solution. SWCNTs possess exceptional electrical conductivity, a high aspect ratio, and a large specific surface area, enabling the formation of an efficient electron transport network within the electrode [25,26]. In addition, their mechanical flexibility helps accommodate structural changes during cycling, thereby improving electrode durability. Recent trends in LIB research have increasingly focused on designing hybrid materials that combine redox-active organic compounds with conductive carbon frameworks to achieve synergistic performance enhancement.

By integrating polymer CoPc (Poly-CoPc) with SWCNTs, it is possible to simultaneously address multiple limitations: improving electrical conductivity, enhancing active material dispersion, increasing electrode–electrolyte contact area, and stabilizing the structure during cycling. Despite these advantages, systematic comparative studies between Poly-CoPc and its SWCNT-modified composites for LIB anode applications remain limited. In this work, we report the synthesis of an oxy-bridged Poly-CoPc and its SWCNT-modified composite (Poly-CoPc/SWCNT), followed by a comprehensive comparative evaluation of their structural and electrochemical properties. This study aims to elucidate the role of SWCNT incorporation in improving charge transport, structural stability, and overall electrochemical performance. The results provide valuable insights into the design of advanced MPc-based hybrid materials for next-generation lithium-ion (Li^+^) battery anodes.

## 2. Experimental Section

### 2.1. Materials

Cobalt (II) chloride hexahydrate (CoCl_2_·6H_2_O), 4,4′-oxydiphthalic anhydride (ODPA, C_16_H_6_O_7_), ammonium chloride (NH_4_Cl), ammonium molybdate tetrahydrate ((NH_4_)_6_Mo_7_O_24_·4H_2_O), and urea (CH_4_N_2_O) were used for Poly-CoPc synthesis. An SWCNT (diameter 1.4–1.7 nm) was used for the modification of the Poly-CoPc/SWCNT composite. Conductive carbon black (Super P) and poly (acrylic acid) (PPA) were used for electrode preparation. Nitrobenzene (C_6_H_5_NO_2_), N-Methyl-2-pyrrolidone (NMP), methanol and ethanol were used as solvents. All chemicals were purchased from Sigma-Aldrich or TCI and used without further purification.

### 2.2. Synthesis of Oxy-Bridged Polymer Cobalt Phthalocyanine (Poly-CoPc)

**Figure 1:**
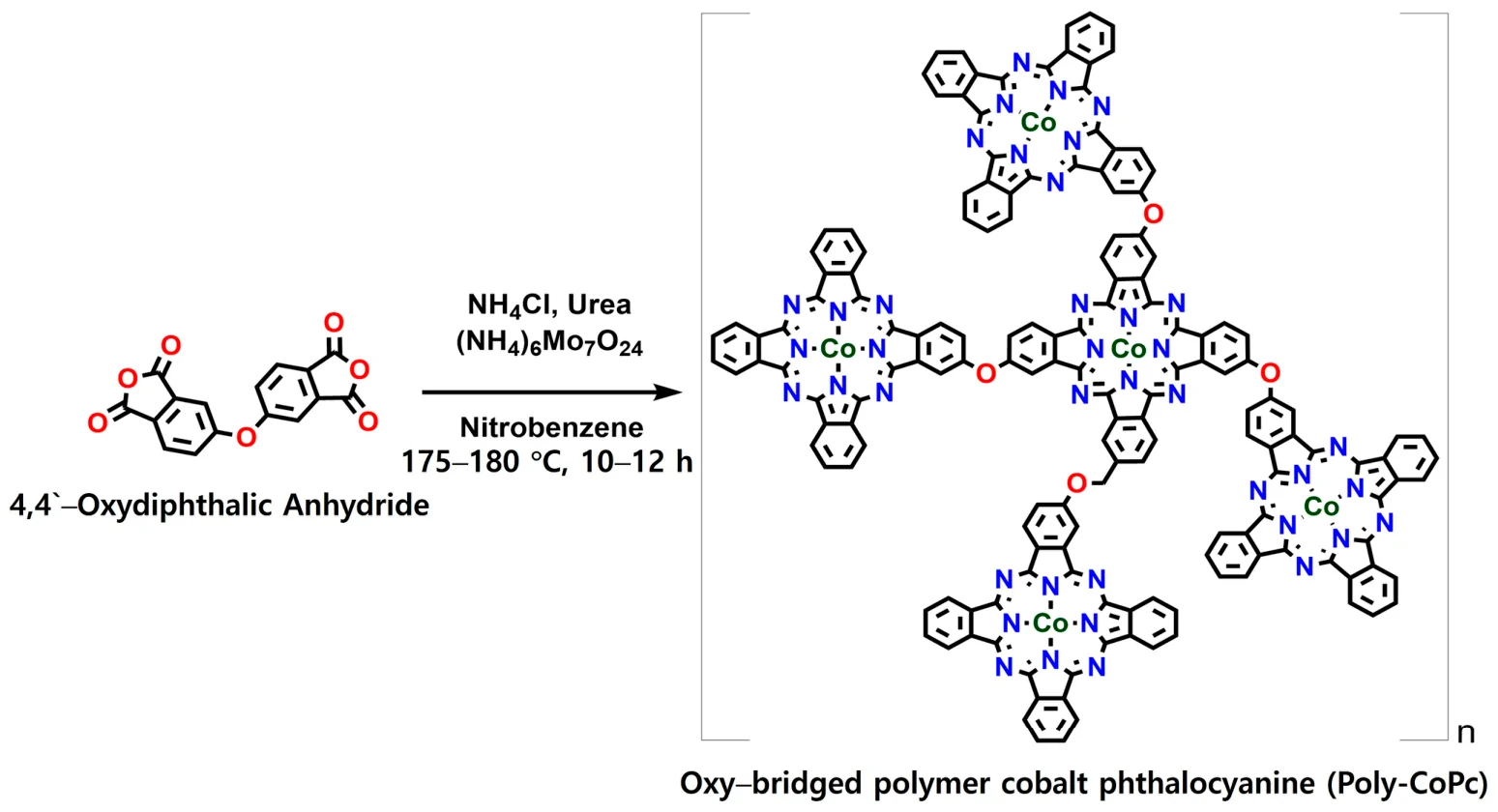
Schematic representation and synthetic routes for Poly-CoPc.

Poly-CoPc was prepared through high-temperature cyclotetramerization followed by polycondensation, as shown in Figure 1, using a method adopted from earlier works [27,28]. In a typical synthesis, cobalt (II) chloride, ODPA, ammonium chloride, and urea were thoroughly ground together in a mortar and pestle in a molar ratio of 2:1:6:12. A small amount of ammonium molybdate was also added as a reaction promotor. The resulting homogeneous mixture was then transferred into a round-bottom flask congaing nitrobenzene and heated to 175–180 °C under a nitrogen atmosphere for 10–12 h with continuous stirring. The completion of the reaction was monitored using TLC. After the completion of the reaction, a dark blue solid product was obtained, collected by filtration and purification by conducting repeated washing with hot water, cold water, ethanol, and acetone to remove unreacted species and residue. Finally, the product was dried under vacuum at 80 °C for 12 h, yielding the cross-linked polymer, referred to as Poly-CoPc.

Yield: 89%. Mol. wt.: [645]_n_. Anal. for Poly-CoPc [C_33_H_14_CoN_8_O_4_]_n_.

### 2.3. Preparation of Oxy-Bridged Poly-CoPc/SWCNT Composite

The oxy-bridged Poly-CoPc/SWCNT composite was prepared by a simple solution-based functionalization method. Briefly, 0.9 mg of Poly-CoPc was dispersed in 10 mL of ethanol by ultrasonication for 30 min. Separately, 0.1 mg of SWCNTs was dispersed in 10 mL of ethanol using probe sonication for 30 min to obtain a homogeneous suspension. The Poly-CoPc dispersion was then added dropwise to the SWCNT suspension under vigorous stirring. The resulting mixture was further stirred at 80 °C for 12 h to promote strong interfacial contact and π–π stacking interactions between Poly-CoPc and SWCNTs. The product was collected by vacuum filtration, washed repeatedly with ethanol and deionized water to remove physically adsorbed species, and dried overnight at 60 °C. The obtained material was denoted as the Poly-CoPc/SWCNT composite.

### 2.4. Characterizations

The morphology and microstructural features of the synthesized Poly-CoPc and Poly-CoPc/SWCNT composite were examined using field-emission scanning electron microscopy (FE-SEM, Hitachi High-Tech Corporation, Toranomon, Japan) and high-resolution transmission electron microscopy (HR-TEM, FEI Tecnai). To further understand the elemental composition and distribution within the composite, energy-dispersive X-ray spectroscopy (EDS) analysis and elemental mapping were carried out using an EDS detector attached to the SEM. The crystalline structure of the materials was analyzed by X-ray diffraction (Bruker D8 Advance X-ray diffractometer). The chemical structure and functional groups were identified using Fourier-transform infrared spectroscopy (FT-IR, Thermo Fisher Scientific iS50) over a wavenumber range of 400–4000 cm^−1^, along with Raman spectroscopy (Olympus BX51 from Smart Materials Core Research Support Center) to gain additional insight into the molecular structure and carbon framework. The specific surface area and pore characteristics of the samples were evaluated using nitrogen adsorption–desorption measurements at 100 K with a Micromeritics ASAP 2020 analyzer.

### 2.5. Electrochemical Measurements

Working electrodes were prepared by thoroughly mixing the active material (Poly-CoPc or Poly-CoPc/SWCNT composite), conductive carbon black (Super P), and poly (acrylic acid) (PAA) binder in a weight ratio of 8:1:1. Ethanol was used as the dispersing solvent to form a uniform slurry. The mixture was stirred until a homogeneous paste was obtained. The prepared slurry was then evenly coated onto a clean copper foil current collector using a doctor blade technique. The coated electrodes were dried in a vacuum oven at 80 °C for 12 h to remove residual solvent and ensure the proper adhesion of the active layer. After drying, the electrodes were punched into circular disks with a diameter of 12 mm, and the degree of active material loading was controlled within the range of approximately 0.85–1.0 mg cm^−2^.

CR2032-type coin cells were assembled in an argon-filled glovebox, where the moisture and oxygen levels were carefully maintained below 0.1 ppm. Li metal foil was used as both the counter and reference electrode. A Celgard microporous polypropylene membrane served as the separator, and the electrolyte consisted of 1 M LiPF_6_ dissolved in a mixture of ethylene carbonate (EC) and diethyl carbonate (DEC) in a 1:1 volume ratio. The electrochemical performance of the assembled cells was evaluated using a WBCS3000 WonAtech (Republic of Korea battery) testing system. Galvanostatic charge–discharge (GCD) measurements were carried out within a voltage range of 0.01–3.0 V (vs. Li^+^/Li) at different current densities to assess the specific capacity, cycling stability, and rate capability of the electrodes. All electrochemical measurements were carried out at room temperature.

## 3. Results and Discussion

### 3.1. Structural and Morphological Characterization

Figure 1 illustrates the synthesis of an oxy-bridged Poly-CoPc starting from 4,4′-oxydiphthalic anhydride as the key precursor. The reaction is carried out under solvothermal conditions using nitrobenzene as the solvent at 185 °C for 10–12 h, in the presence of NH_4_Cl, urea, and ammonium molybdate ((NH_4_)_6_Mo_7_O_24_) as auxiliary reagents. Initially, 4,4′-oxydiphthalic anhydride undergoes a series of condensation and cyclization reactions with nitrogen-containing species generated from urea and ammonium salts. These reactions lead to the formation of the phthalocyanine macrocyclic ring, where cobalt ions are centrally coordinated by four isoindole nitrogen atoms, resulting in the formation of Co–N_4_ units. A key feature of this synthesis is the presence of the –O–(oxy) bridge within the precursor molecule. This oxygen linkage plays a crucial role in connecting multiple phthalocyanine units, leading to the formation of an extended polymeric network rather than isolated monomeric CoPc molecules. As a result, individual CoPc units are covalently linked through aromatic segments, producing a three-dimensional or chain-like conjugated polymer structure. This polymeric architecture offers several important advantages such as enhanced structural stability compared to monomeric CoPc, reducing dissolution during electrochemical cycling; extending π-conjugation, which facilitates better electron delocalization and the presence of multiple redox-active Co–N_4_ centers; increasing the number of active sites and forming a porous and interconnected network, beneficial for electrolyte penetration. Moreover, the oxygen bridges introduce structural flexibility and spacing between CoPc units, preventing dense stacking and improving the accessibility of Li^+^.

**Figure 2:**
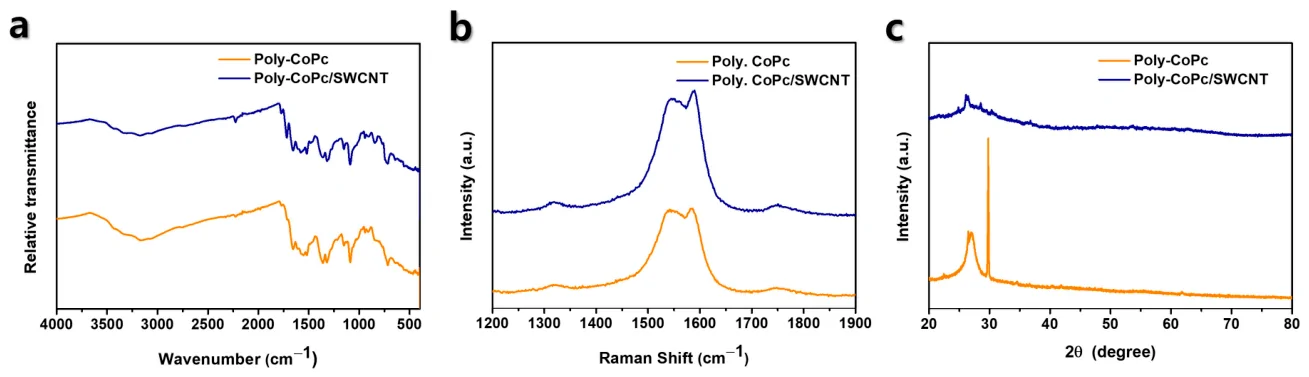
Characterization of both Poly-CoPc and Poly-CoPc/SWCNT composite. (**a**) FT-IR spectra were recorded from 4000 to 400 cm^−1^. (**b**) Raman spectra. (**c**) PXRD patterns were scanned over 2θ range of 20° to 80° with counting time of 1 s per step.

The formation of Poly-CoPc and its successful integration with SWCNTs were first confirmed using FT-IR and Raman spectroscopy (Figure 2). In Figure 2a, the FT-IR spectra of Poly-CoPc display characteristic bands corresponding to the phthalocyanine macrocyclic structure. The prominent peaks observed in the ~1600–1500 cm^−1^ region are attributed to the C=N and C=C stretching vibrations of the conjugated aromatic system, while bands around ~1300–1100 cm^−1^ correspond to C–N stretching, confirming the formation of the coordinated CoPc framework. In the case of the Poly-CoPc/SWCNT composite, these characteristic peaks are retained but show slight shifts and reduced intensity, indicating strong interfacial interaction between Poly-CoPc and SWCNTs. The broadening of these bands suggests the improved dispersion of the polymer over the nanotube surface and partial structural disorder induced by the incorporation of SWCNTs. Raman spectroscopy further supports these observations (Figure 2b). The composite exhibits the typical D-band (~1320 cm^−1^) and G-band (~1565 cm^−1^) associated with graphitic carbon. The increased intensity ratio (I_D_/I_G_) in the composite indicates the introduction of defects and edge sites, which are beneficial for electrochemical reactions. These changes confirm that SWCNTs are well integrated with Poly-CoPc and contribute to enhanced electrical conductivity and active site availability.

The XRD patterns provide insight into the structural arrangement of the materials (Figure 2c). Poly-CoPc shows distinct diffraction peaks at 2θ ≈ 26.95° and 29.84°, indicating a partially crystalline structure. The peak near 26.95° can be attributed to π–π stacking interactions between phthalocyanine rings, while the peak at 29.84° corresponds to ordered molecular packing within the polymer network. Upon the incorporation of SWCNTs, the diffraction peaks become broader and less intense, particularly in the 25–28° region. This broad feature is characteristic of the graphitic (002) plane of SWCNTs. The reduction in peak intensity indicates a decrease in crystallinity, suggesting that SWCNTs disrupt the long-range order of Poly-CoPc and promote a more amorphous, well-dispersed structure. Such a structure is advantageous for electrochemical applications, as it enhances electrolyte accessibility and ion transport.

**Figure 3:**
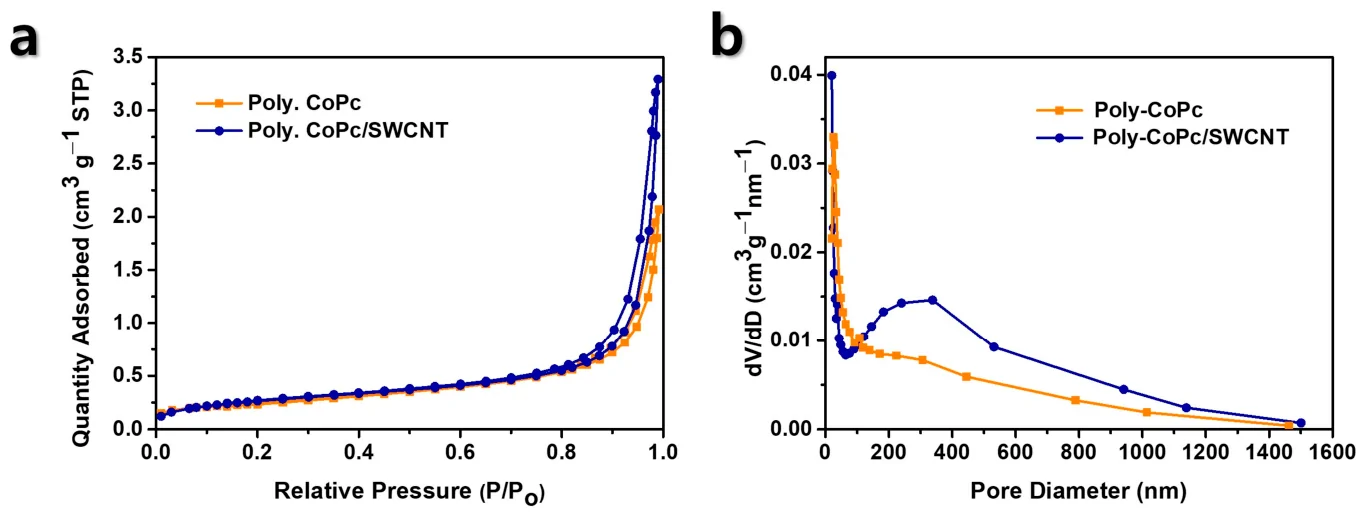
(**a**) BET surface area analysis derived from N_2_ adsorption–desorption isotherms of Poly-CoPc and Poly-CoPc/SWCNT. (**b**) Pore size distribution of Poly-CoPc and Poly-CoPc/SWCNT.

**Figure 4:**
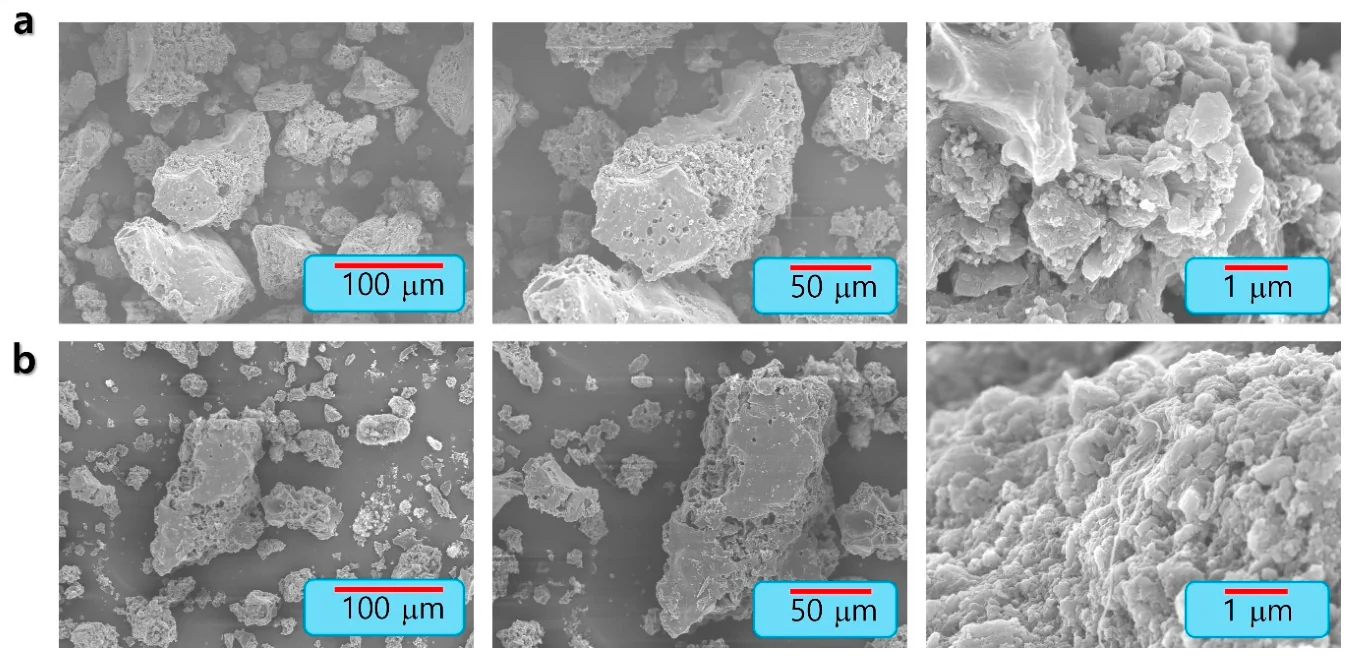
SEM images of (**a**) Poly-CoPc and (**b**) Poly-CoPc/SWCNT materials.

As shown in Figure 3 and Table 1, the BET results show that the Poly-CoPc/SWCNT composite has a higher surface area (31.29 m^2^ g^−1^) and larger pore volume (0.0958 cm^3^ g^−1^) than pristine Poly-CoPc (19.17 m^2^ g^−1^ and 0.0624 cm^3^ g^−1^). This indicates that the incorporation of SWCNTs creates a more open and porous structure, which improves electrolyte access and Li^+^ transport. As a result, the composite provides more active sites and better electrochemical utilization, contributing to its enhanced battery performance. The surface morphology and microstructural features of Poly-CoPc and the Poly-CoPc/SWCNT composite were examined using SEM at different magnifications (Figure 4). At lower magnification (100 μm and 50 μm scale), the pristine Poly-CoPc exhibits irregular, block-like particles with relatively large sizes and non-uniform distribution. These particles appear densely packed and aggregated, indicating that the polymer chains tend to cluster together during synthesis. The surfaces of these particles are relatively rough and compact, which can limit electrolyte penetration and reduce the number of accessible active sites for Li^+^ storage. As the magnification increases (1 μm scale), the structure of Poly-CoPc becomes clearer, revealing coarse, stacked domains with limited porosity. This dense morphology suggests restricted ion diffusion pathways and poor interfacial contact with the electrolyte, which can negatively affect electrochemical performance. In contrast, the Poly-CoPc/SWCNT composite shows a significantly different morphology. At similar magnifications, the composite displays a more fragmented and loosely packed structure, with smaller particle sizes and improved dispersion. The presence of SWCNTs disrupts the dense aggregation of Poly-CoPc and leads to the formation of a more open and interconnected network.

**Figure 5:**
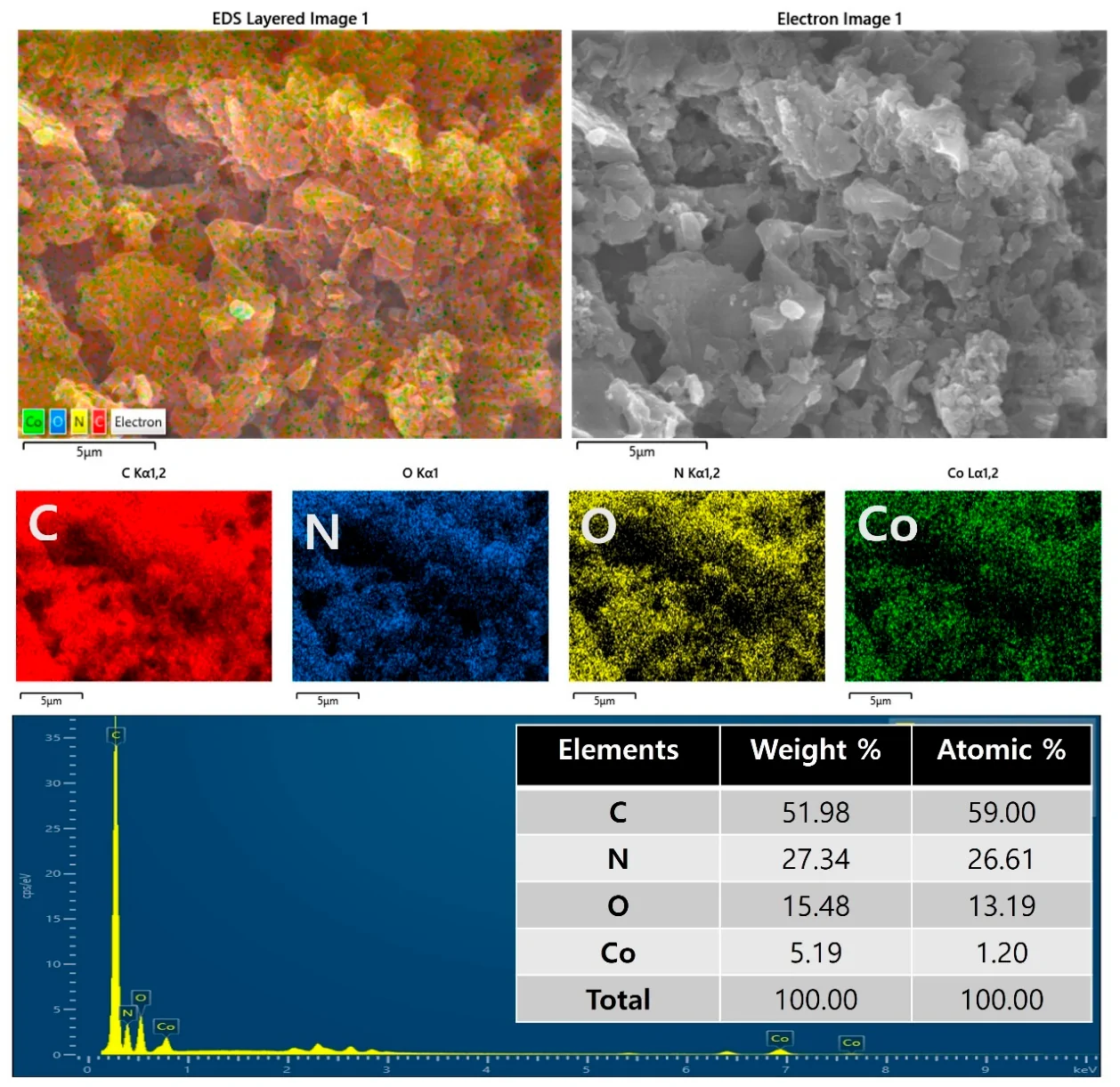
SEM-EDX images of Poly-CoPc/SWCNT.

To further confirm the elemental composition and distribution, SEM–EDX mapping was performed for the Poly-CoPc/SWCNT composite (Figure 5). The elemental maps show the presence of carbon (C), nitrogen (N), oxygen (O), and cobalt (Co) distributed throughout the sample. C is uniformly distributed, originating from both the phthalocyanine framework and the SWCNTs, and N is evenly dispersed, confirming the presence of the Co–N_4_ coordination structure in the phthalocyanine ring. Similarly, O is distributed across the sample, consistent with the oxy-bridged polymer structure. Finally, cobalt is homogeneously dispersed without noticeable clustering, indicating the successful incorporation of the metal centers within the polymer framework. The uniform distribution of all elements suggests that the composite has a well-integrated and homogeneous structure, which is essential for consistent electrochemical activity.

**Figure 6:**
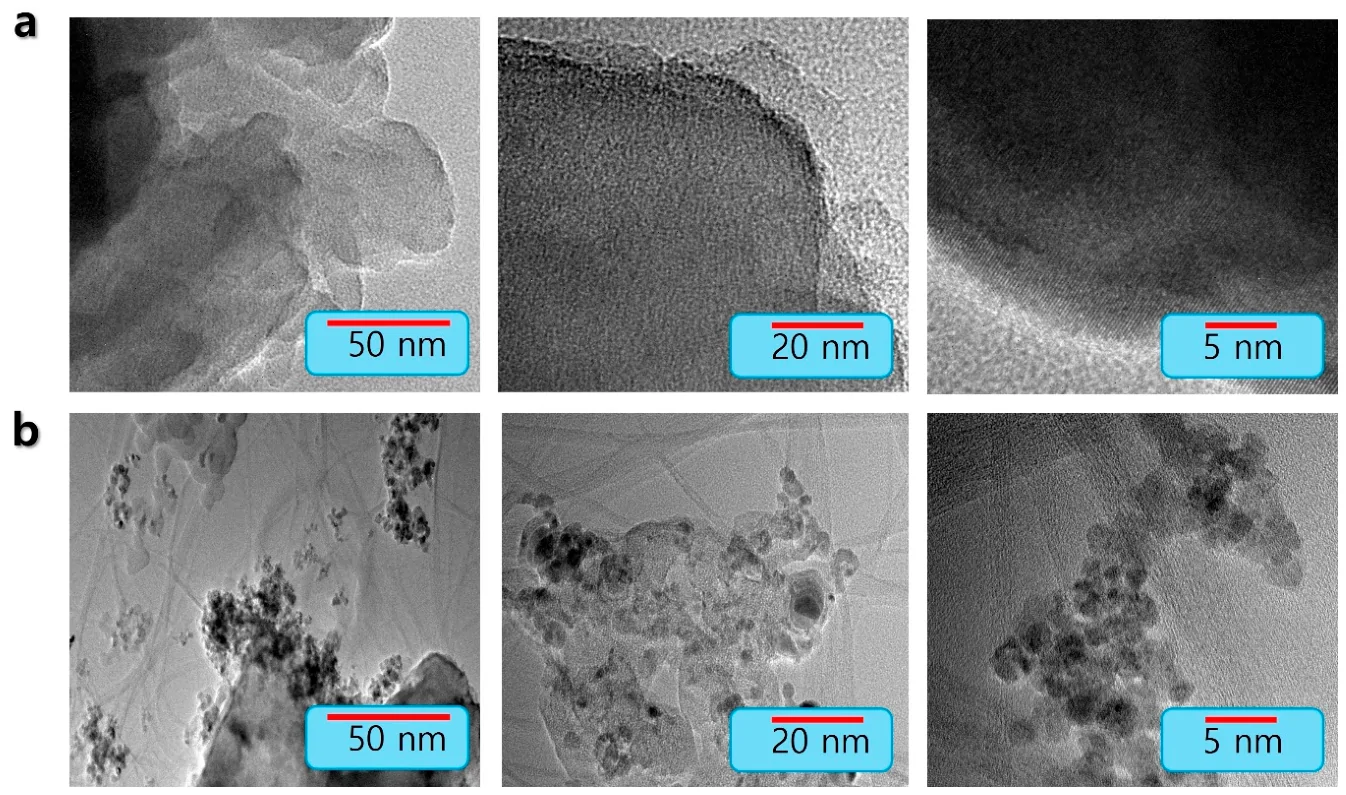
TEM images of (**a**) Poly-CoPc and (**b**) Poly-CoPc/SWCNT materials.

The detailed surface morphologies of Poly-CoPc and the Poly-CoPc/SWCNT composite were further examined using transmission electron microscopy (TEM) at different magnifications (Figure 6). For the pristine Poly-CoPc, the TEM images (50 nm to 5 nm scale) reveal relatively thick, sheet-like and aggregated structures. These layers appear dense and somewhat featureless at lower magnification, indicating that the polymer forms stacked domains with limited internal porosity. At higher magnification (20 nm and 5 nm), the structure shows a compact arrangement with weakly defined boundaries, suggesting restricted pathways for Li^+^ diffusion. This observation is consistent with the SEM and BET results, where Poly-CoPc exhibited a relatively dense morphology and moderate surface area. In contrast, the Poly-CoPc/SWCNT composite shows a significantly different and more favorable nanostructure. At lower magnification, a network-like structure composed of thin, interconnected nanotubes is clearly visible, confirming the presence of SWCNTs. Poly-CoPc appears to be uniformly anchored onto and wrapped around the SWCNT framework, forming a well-integrated hybrid structure. At intermediate magnification (20 nm scale), the composite exhibits finely distributed nanostructured domains, where small particles or clusters of Poly-CoPc are attached along the nanotube surfaces. This uniform dispersion prevents large-scale aggregation and increases the exposure of active sites. At higher magnification (5 nm scale), the TEM images reveal highly dispersed nanoscale clusters embedded within or attached to the SWCNT network. The presence of these fine features suggests that Poly-CoPc is present in a nanostructured form with reduced particle size, which is highly beneficial for electrochemical applications. In addition, faint lattice-like fringes associated with graphitic carbon can be observed, confirming the structural integrity of SWCNTs within the composite.

### 3.2. Electrochemical Performance Evaluation

**Figure 7:**
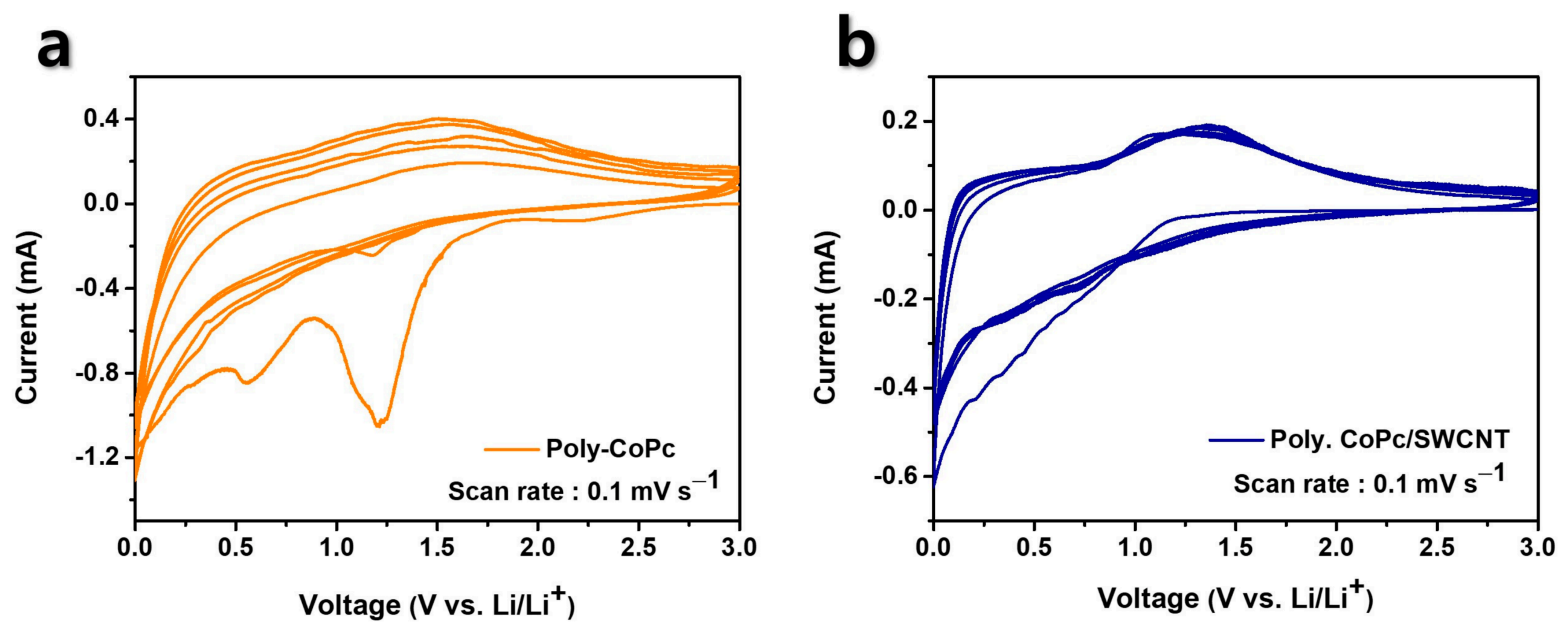
(**a**) Cyclic voltammetry (CV) curves of Poly-CoPc electrode at scan rate of 0.1 mV s^−1^ in initial five cycles. (**b**) CV curves of Poly-CoPc/SWCNT composite electrode at scan rate of 0.1 mV s^−1^ in initial five cycles.

The electrochemical behavior of Poly-CoPc and the Poly-CoPc/SWCNT composite was investigated using CV at 0.1 mV s^−1^ scan rates (Figure 7). The CV curves provide valuable insight into the redox activity, charge storage mechanism, and kinetic properties of the electrode materials. For pristine (Figure 7a) Poly-CoPc, the CV curves exhibit noticeable redox peaks, indicating that Li storage occurs through faradaic reactions associated with the Co–N_4_ active centers and the conjugated phthalocyanine framework. However, the redox peaks appear relatively broad and less defined, suggesting sluggish reaction kinetics and higher polarization. As the scan rate increases, a significant distortion in the CV shape is observed, which reflects limited charge transfer efficiency and slower ion diffusion within the polymer structure. In contrast, the Poly-CoPc/SWCNT composite (Figure 7b) shows a much more stable and well-defined CV profile. The redox peaks are sharper and more symmetric, indicating the enhanced reversibility of the electrochemical reactions. Additionally, the peak currents are significantly higher compared to pristine Poly-CoPc, demonstrating improved electrochemical activity and the better utilization of active sites.

**Figure 8:**
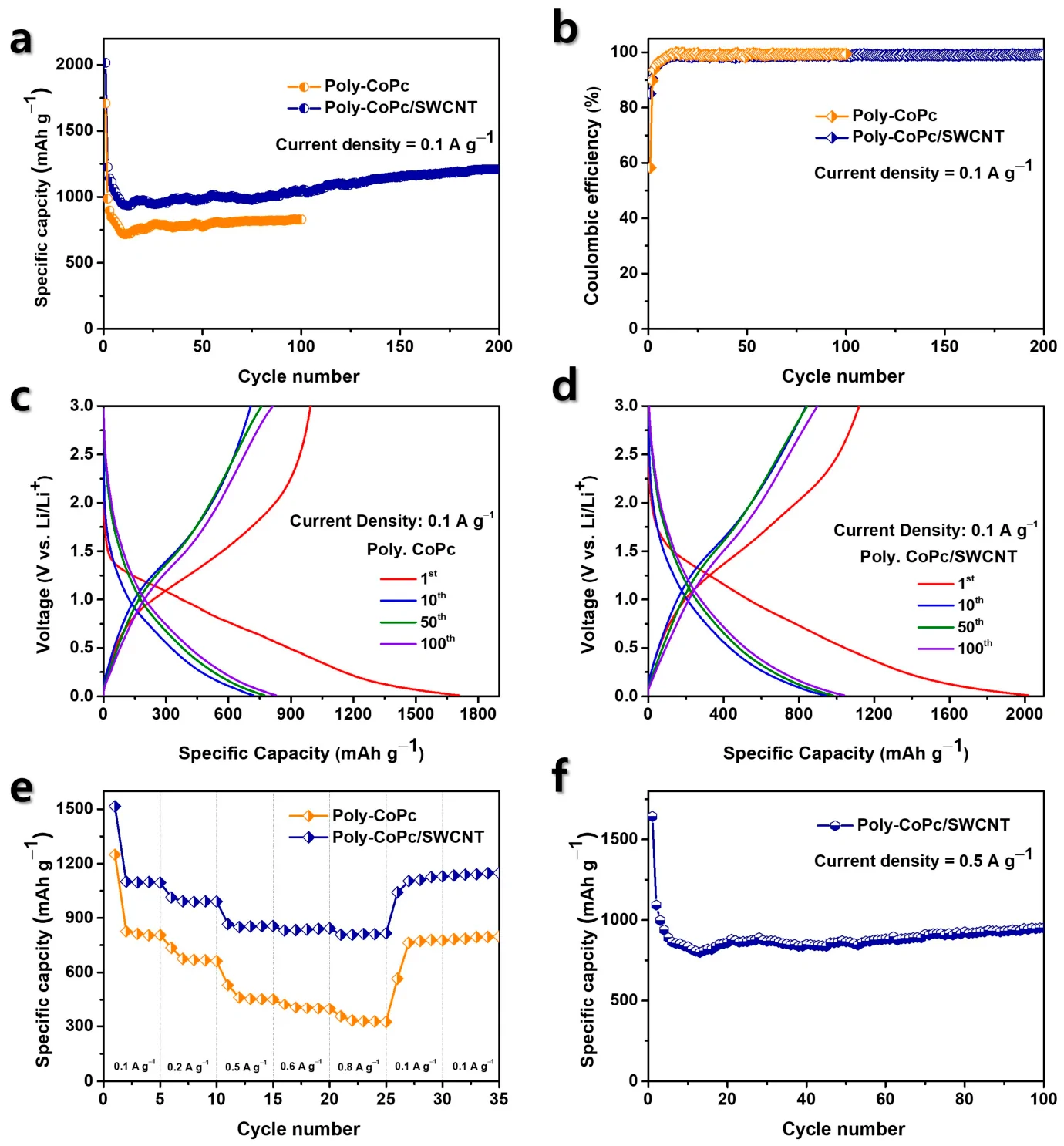
(**a**) Cycling performances of Poly-CoPc and Poly-CoPc/SWCNT at 0.1 A g^−1^. (**b**) Coulombic efficiency of Poly-CoPc and Poly-CoPc/SWCNT at 0.1 A g^−1^. (**c**) GCD profile for Poly-CoPc at 0.1 A g^−1^. (**d**) GCD profile for Poly-CoPc/SWCNT at 0.1 A g^−1^. (**e**) Rate capacity of both Poly-CoPc and Poly-CoPc/SWCNT electrodes at different current densities. (**f**) Cycling performance of Poly-CoPc/SWCNT at higher current density of 0.5 A g^−1^.

Figure 8a and Table 2 shows the cycling performance of Poly-CoPc and Poly-CoPc/SWCNT at 0.1 A g^−1^, and the cycling stability clearly highlights the advantage of incorporating SWCNTs. The pristine Poly-CoPc electrode shows an initial discharge capacity of 1703 mA g^−1^, which decreases to 803 mA g^−1^ after 100 cycles. By contrast, the Poly-CoPc/SWCNT composite delivers a higher initial discharge capacity of 2016 mA g^−1^ and retains a capacity of 1047 mA g^−1^ after 100 cycles. This level of capacity retention demonstrates excellent long-term stability. In contrast, pristine Poly-CoPc shows a rapid capacity decay during the initial cycles and stabilizes at a significantly lower capacity. This behavior is mainly due to structural degradation, poor electrical conductivity and the limited accessibility of active sites. The improved cycling stability of the composite can be attributed to the robust hybrid structure, where SWCNTs act as a flexible conductive backbone that accommodates volume changes during repeated lithiation and delithiation. Figure 8b illustrates the Coulombic efficiency of Poly-CoPc and Poly-CoPc/SWCNT at 0.1 A g^−1^. The Poly-CoPc/SWCNT electrode shows a higher initial Coulombic efficiency (85.06%) than Poly-CoPc (58.30%), indicating lower irreversible capacity loss in the first cycle (Table 3). Both electrodes gradually stabilize with cycling and reach nearly 100% Coulombic efficiency, confirming good reversibility and stable electrochemical performance.

The galvanostatic charge–discharge (GCD) profiles of both Poly-CoPc and the Poly-CoPc/SWCNT composite at 0.1 A g^−1^ are shown in Figure 8c,d respectively to evaluate their Li storage behavior. In the initial cycles, both materials exhibit a high discharge capacity, which is commonly associated with the formation of a solid electrolyte interphase (SEI) layer and irreversible side reactions. However, a clear distinction is observed between the two materials after the initial cycles. The Poly-CoPc/SWCNT composite shows a much higher initial discharge capacity compared to pristine Poly-CoPc, indicating the improved electrochemical utilization of active sites. More importantly, from the second cycle onward, the charge–discharge curves of the composite become highly stable and reproducible, suggesting good reversibility and structural stability. The voltage profiles of the composite are smoother and exhibit reduced polarization compared to Poly-CoPc. This indicates lower internal resistance and faster reaction kinetics, which can be attributed to the conductive SWCNT network facilitating efficient electron transport.

Figure 8e shows the rate performance of Poly-CoPc and Poly-CoPc/SWCNT electrodes at various current densities, and the corresponding rate capacity comparison is presented in Table 4. The rate capability results clearly demonstrate the superior kinetic performance of the composite. As current density increases, both materials show a decrease in capacity; however, the composite retains significantly higher capacity at all current densities. Notably, the Poly-CoPc/SWCNT composite delivers a capacity of 855 mA g^−1^ at 0.5 A g^−1^, indicating excellent rate capability. When current density is reduced back to lower values, the composite recovers its capacity effectively, confirming its structural robustness and reversibility. At high current densities, the composite continues to perform efficiently, maintaining stable capacity with minimal polarization. This behavior indicates fast electron transport and rapid Li^+^ diffusion, which are essential for high-power applications. Figure 8f presents the cycling stability of the Poly-CoPc/SWCNT electrode at a high current density of 0.5 A g^−1^. Overall, the Poly-CoPc/SWCNT composite demonstrates remarkably enhanced Li storage performance compared to pristine Poly-CoPc. The combination of high capacity, excellent cycling stability, and strong rate capability makes this composite a highly promising candidate for advanced Li^+^ battery anodes. Table 5 summarizes the comparative electrochemical performance of Poly-CoPc and Poly-CoPc/SWCNT with other previously reported anode materials.

**Figure 9:**
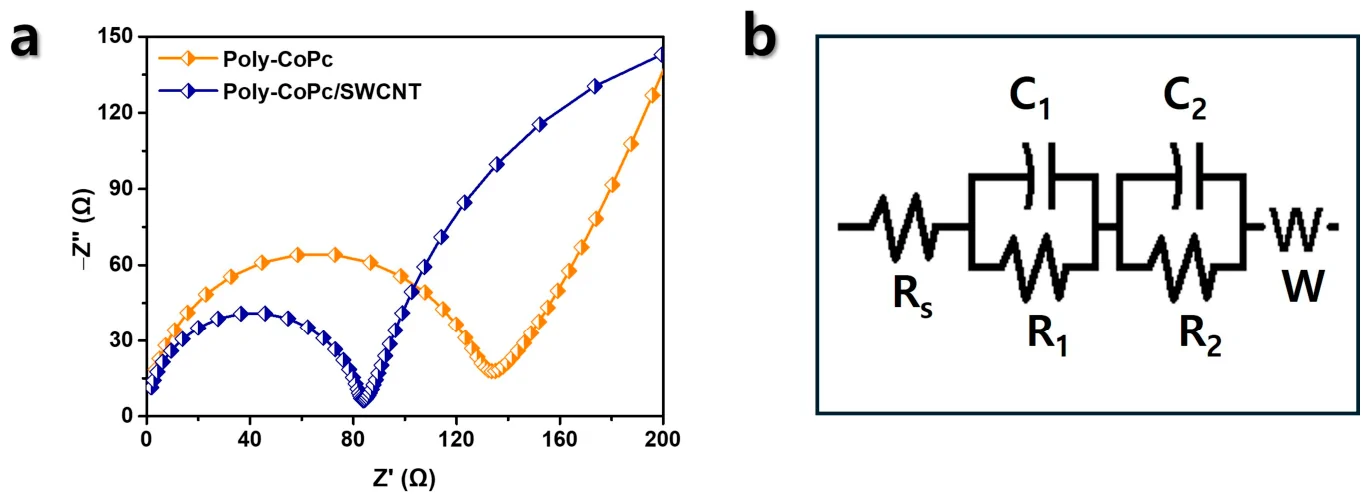
(**a**) EIS Nyquist plots after 30 cycles for Poly-CoPc and Poly-CoPc/SWCNT; (**b**) shows the equivalent circuit used to fit the EIS Nyquist plots after 30 cycles for Poly-CoPc and Poly-CoPc/SWCNT.

Electrochemical impedance spectroscopy (EIS) was carried out to investigate the interfacial charge transfer and Li^+^ diffusion behavior of the electrodes. Figure 9 shows the EIS Nyquist plots of Poly-CoPc and Poly-CoPc/SWCNT after 30 cycles. Nyquist plots were fitted using an equivalent circuit consisting of R_s_, R_1_/C_1_, R_2_/C_2_, and a Warburg diffusion element (W) (Figure 9b). Table 6 lists the fitted equivalent circuit parameters for the modified electrodes. Compared with pristine Poly-CoPc, the Poly-CoPc/SWCNT composite exhibits substantially lower R_1_ (15.55 Ω) and R_2_ (124.2 Ω) values than Poly-CoPc (81.23 Ω and 220.1 Ω, respectively), indicating reduced interfacial resistance and faster charge transfer kinetics after SWCNT incorporation. In addition, the lower W value of the composite (0.001018 Ω) compared to Poly-CoPc (0.004852 Ω) suggests improved Li^+^ diffusion. These results confirm that the conductive SWCNT network effectively enhances electron transport, stabilizes the electrode/electrolyte interface, and promotes ion diffusion, which together contribute to the superior electrochemical performance of the Poly-CoPc/SWCNT electrode.

Overall, the present study demonstrates that incorporating SWCNTs into the Poly-CoPc matrix significantly improves electrochemical performance through enhanced conductivity, improved charge transport, and increased structural stability. The synergistic interaction between the redox-active polymer and conductive SWCNT network resulted in improved reversible capacity, excellent cycling stability, and enhanced rate capability. These findings provide valuable insight into the design strategy of polymer–carbon hybrid materials and suggest that the Poly-CoPc/SWCNT composite has strong potential as a promising anode material for next-generation high-performance lithium-ion batteries.

## 4. Conclusions

Poly-CoPc and its SWCNT-modified composite were successfully synthesized and evaluated as anode materials for LIBs. Comprehensive characterization (FT-IR, Raman, XRD, BET, SEM, and TEM) confirmed the formation of the polymer framework and its effective integration with the SWCNT network, leading to improved surface area, porosity, and morphology. The electrochemical results show that the Poly-CoPc/SWCNT composite delivers significantly enhanced performance compared to pristine Poly-CoPc, including a high initial capacity of 2016 mA g^−1^, excellent capacity retention of 1047 mA g^−1^ after 100 cycles at 0.1 A g^−1^, and strong rate capability (855 mA g^−1^ at 0.5 A g^−1^). These improvements are supported by EIS analysis, which indicates reduced charge transfer resistance and faster Li^+^ diffusion. The superior performance of the composite is attributed to the synergistic combination of the redox-active polymer and the highly conductive SWCNT network, which enhances electron transport, improves active material dispersion, and facilitates efficient ion diffusion. Overall, this study highlights that integrating Poly-CoPc with conductive carbon nanostructures is an effective approach for designing high-performance anode materials, making the Poly-CoPc/SWCNT composite a promising candidate for next-generation LIBs.

## Figures and Tables

**Table 1:** Surface area and pore volume measured by BET for Poly-CoPc and Poly-CoPc/SWCNT electrode.

Material	Surface Area	Pore Volume
Poly-CoPc	19.17 m^2^ g^−1^	0.0624 cm^3^ g^−1^
Poly-CoPc/SWCNT	31.29 m^2^ g^−1^	0.0958 cm^3^ g^−1^

**Table 2:** Performance comparison of Poly-CoPc and Poly-CoPc/SWCNT electrode materials.

Materials	Initial Discharge Capacity (mA g^−1^)	Discharge Capacity After 100 Cycles (mA g^−1^)	Capacity Retention After 100 Cycles (%)
Poly-CoPc	1703	803	47.15
Poly-CoPc/SWCNT	2016	1047	51.93

**Table 3:** Coulombic efficiency of Poly-CoPc and Poly-CoPc/SWCNT electrode at current density of 0.1 A g^−1^ for initial 10 cycles.

Cycle Number	Coulombic Efficiency (%)	
	Poly-CoPc	Poly-CoPc/SWCNT
1st	58.29749	85.06311
2nd	89.78123	90.44424
3rd	93.66723	93.8655
4th	95.29852	94.71627
5th	96.40965	95.84065
6th	96.76784	96.26624
7th	97.39316	97.06243
8th	97.69082	97.35259
9th	98.45782	97.72279
10th	98.60257	98.31385

**Table 4:** A comparison of the rate capacity of Poly-CoPc and the Poly-CoPc/SWCNT electrode.

Rate Capacity [mAh g^−1^]	0.1 A g^−1^	0.2 A g^−1^	0.5 A g^−1^	0.6 A g^−1^	0.8 A g^−1^	0.1 A g^−1^	Regained Capacity (%)
Poly-CoPc	804	667	453	396	332	777	96.64
Poly-CoPc/SWCNT	1102	989	847	831	812	1118	101.45

**Table 5:** Comparison study of Poly-CoPc and Poly-CoPc/SWCNT with other previously reported materials.

Samples	Cycles	Current Density (mA g^−1^)	Discharge Capacity (mA H g^−1^)	Reference
SnO2/N-PC	1000	200	775	[29]
Fe2O3/N-PC			612	
CoPc	100	100	327	[30]
CoPc/MWCNT			488	
Carbon-coated Ga_2_S_3_	100	200	642	[31]
Cu_6_Sn_5_/C	100	100	479	[32]
CoTAPc-PDA	100	300	602	[33]
CoTAPc-BDA			701	
CoTAPc-TDA			865	
Ti_3_C_2_/NiCoP	100	100	378	[34]
PANI/SWCNT	100	100	830	[35]
CoPc@CNF	1200	500	740	[36]
A-CuTCAPc/SWCNT	100	100	810	[37]
CuTAPc	50	200	236	[38]
MA–VA–PcNi polymer	200	400	507	
NiPc	100	100	255	[39]
NiPc/SWCNT			703	
Poly-CoPc	100	100	803	This work
Poly-CoPc/SWCNT			1047	

**Table 6:** Equivalent circuit parameters for the modified electrodes.

Electrode	R_s_ (Ω)	R_1_ (Ω)	R_2_ (Ω)	C_1_ (F)	C_2_ (F)	W (Ω)
Poly-CoPc	0.01	81.23	220.1	1.386 × 10^−7^	0.0003752	0.004852
Poly-CoPc/ SWCNT	0.1	15.55	124.2	0.0001755	8.892 × 10^−8^	0.001018

## Data Availability

The original contributions presented in this study are included in the article. Further inquiries can be directed to the corresponding authors.
